# Altering APP Proteolysis: Increasing sAPPalpha Production by Targeting Dimerization of the APP Ectodomain

**DOI:** 10.1371/journal.pone.0040027

**Published:** 2012-06-29

**Authors:** Clare A. Peters Libeu, Olivier Descamps, Qiang Zhang, Varghese John, Dale E. Bredesen

**Affiliations:** Buck Institute for Research on Aging, Novato, California, United States of America; Thomas Jefferson University, United States of America

## Abstract

One of the events associated with Alzheimer's disease is the dysregulation of α- versus β-cleavage of the amyloid precursor protein (APP). The product of α-cleavage (sAPPα) has neuroprotective properties, while Aβ1-42 peptide, a product of β-cleavage, is neurotoxic. Dimerization of APP has been shown to influence the relative rate of α- and β- cleavage of APP. Thus finding compounds that interfere with dimerization of the APP ectodomain and increase the α-cleavage of APP could lead to the development of new therapies for Alzheimer's disease. Examining the intrinsic fluorescence of a fragment of the ectodomain of APP, which dimerizes through the E2 and Aβ-cognate domains, revealed significant changes in the fluorescence of the fragment upon binding of Aβ oligomers—which bind to dimers of the ectodomain— and Aβ fragments—which destabilize dimers of the ectodomain. This technique was extended to show that RERMS-containing peptides (APP_695_ 328–332), disulfiram, and sulfiram also inhibit dimerization of the ectodomain fragment. This activity was confirmed with small angle x-ray scattering. Analysis of the activity of disulfiram and sulfiram in an AlphaLISA assay indicated that both compounds significantly enhance the production of sAPPα by 7W-CHO and B103 neuroblastoma cells. These observations demonstrate that there is a class of compounds that modulates the conformation of the APP ectodomain and influences the ratio of α- to β-cleavage of APP. These compounds provide a rationale for the development of a new class of therapeutics for Alzheimer's disease.

## Introduction

Alzheimer's disease (AD) affects more than five million people in the US, yet it is currently without any truly effective treatment. Although interest has in the past focused primarily on amyloid-β (Aβ), both soluble and plaque-associated, previous research from our laboratory and others has shown that, at least in transgenic mouse models of AD, high levels of Aβ and plaque formation do not necessarily lead to the Alzheimer's phenotype [1]. Instead, both pre-plaque pathophysiology—possibly due to APP signaling and including Aβ oligomers [2], [3], [4] —and distinct, plaque-related pathology appear to be involved.

A number of previous reports have demonstrated toxicity of Aβ based on chemical and physical effects on the cell, such as lysosomatropic detergent-like effects [5], metal binding [6], generation of reactive oxygen species [7], and inhibition of LTP [8]. Recent, complementary results argue that signaling events, some of which are mediated by the amyloid precursor protein (APP) itself, also play a crucial role in the development of the AD phenotype [2], [9], [10], [11], [12], [13]. Furthermore, APP has recently been shown to be a receptor for netrin-1 [13], an axon guidance and survival factor and to give rise to N-APP, a ligand for DR6 (death receptor 6) [14]. In addition, binding of the multimeric APP to Aβ oligomers has recently been shown to promote the proteolytic processing of APP and increase the production of the toxic Aβ and C-31 peptides [15]. Thus, APP may mediate either trophic, anti-apoptotic events—when bound, for example, by netrin-1—or anti-trophic, pro-apoptotic events—when bound, for example, by Aβ [15], [16], [17]. Aβ-binding to APP is not well understood at the structural level. We have recently shown that Aβ oligomers bind to parallel dimers of purified APP ectodomain and effect a distinct conformational change—movement of the E1 domain away from the E2 domain—that can be detected by small angle x-ray scattering (SAXS); conversely, Aβ dimers bind to parallel dimers of APP ectodomain and split the APP ectodomain homodimer, as well as opening the compact amino-terminal structure (“popping the top”) of the APP ectodomain [18].

These changes in conformation may be important in setting the balance between α-cleavage and β-cleavage of APP because the relative rates of these cleavages are known to be affected by interactions between APP and a number of proteins, both at the cell surface and during recycling of APP [19], [20]. Altering the balance of these two cleavages to favor α-cleavage is of potential therapeutic benefit for Alzheimer's disease since it would reduce Aβ production and enhance sAPPα production, without inhibition of the beta and gamma secretases. A precedent exists for this effect in that an increased level of sAPPα, which contains almost the entire ectodomain, has been shown to inhibit APP dimerization and increase cell survival [21]. Enforced dimerization of APP through the E1 domain or the Aβ-cognate region with engineered disulfides has been shown to increase Aβ production, while forced dimerization through the transmembrane domain or the C-terminal domain [22], [23] can reduce Aβ production. These results suggest that the conformation of APP in the dimer and the mechanism of dimerization may play an important role in APP processing. Since APP is regulated through interactions with a number of different proteins, forcing dimerization may expose specific binding sites within the APP ectodomain and thereby influence its ultimate fate.

Models of APP dimerization have been proposed based on the dimerization properties of truncation fragments or APP proteins overexpressed with deleted domains in which the E1 domain plays a major role [24]. Purified fragments containing either the E1 domain or its subdomains or the E2 have also been shown to dimerize with and without disulfide cross-links, suggesting that there may be multiple ways for APP fragments to dimerize given sufficient concentration [25], [26], [27]. However, sAPPα, which contains both the E1 and E2 domains, is a monomer in solution and requires the presence of heparin to dimerize or form heterocomplexes with APP [21], [24], [28]. In contrast, fragments of the APP ectodomain containing the last nine residues of the APP ectodomain (residues 615–624) that are not found in sAPPα form parallel dimers through the E2 domain and Aβ-cognate region independent of the presence of the E1 domain, suggesting that the E2 domain and the Aβ-cognate region may play important roles in stabilizing dimers of the full-length APP [18]. The inconsistency between the different models of APP dimers may lie in the biology of APP in that the APP molecules are anchored on the cell surface through their transmembrane domains; therefore, APP molecules on the same cell are restricted to a parallel orientation, while sAPPα and the other ectodomain fragments are free to bind in both parallel and anti-parallel orientations.

The location of the two potential dimer interfaces of the purified ectodomain of APP are particularly interesting because these locations are close to the α- and β- cleavage sites and one of the major heparin-binding sites [29]. Compounds that disrupt these dimerization interfaces might be able to alter the conformation of the ectodomain sufficiently to alter the conformation of APP adjacent to the α- and β- cleavage sites (and therefore alter the balance of α- and β- cleavage directly), interfere with the formation of heterodimers of APP, or interfere with dimerization of APP. In the current study, we tested the feasibility of using the intrinsic fluorescence of a fragment of the ectodomain of APP (residues 230–624), which contains both dimerization interfaces, to identify molecules that can inhibit dimerization of the ectodomain of APP. We show that two compounds with a similar quenching effect on the intrinsic fluorescence bind to the same fragment of APP and modify the proteolysis of intact APP to favor the production of sAPPα. These results suggest that compounds that inhibit the dimerization of the ectodomain of APP may increase sAPPα production, thus providing a rationale for the development of a new class of therapeutics for Alzheimer's disease. Herein we have identified two such compounds that bring about an increase in sAPPα with a concomitant decrease in sAPPβ through inhibition of the dimerization of the ectodomain of APP.

## Methods

### Reagents

Dulbecco's phosphate buffered saline (PBS) was purchased from MediaTech. Disulfiram and Sulfiram were purchased from Tocris Biosciences, 1-anilinonaphthalene-8-sulfonic acid (1,8-ANS) was purchased from Invitrogen. ADAM10 was purchased from Sigma.

### Reconstitution of the peptides and preparation of the oligomers

All peptides were purchased from Anaspec. All of the work reported herein was performed using one peptide lot. The APP319-335 peptide and RERMS peptides were reconstituted in PBS. The Aβ peptides were reconstituted by adding 30 µl of 100 mM sodium hydroxide to 0.5 mg of peptide. Once the peptides were fully dissolved, PBS was added to bring the concentration to 10–20 µM for Aβ1-40 or Aβ1-42. The more soluble Aβ fragments were diluted to 200 µM. The preparation of the Aβ oligomers is extensively described in Libeu et al (2011) [18] which shows that this method produces three distinct size-ranges of oligomers: 7-kDa dimers, a 13–20-kDa mixture of trimers to hexamers and a 70-kDa population of primarily dodecamers. In short, the aliquots of Aβ1-40 or Aβ1-42 were incubated at 37°C for variable times and then characterized using a calibrated Superdex 75 column with PBS as the running buffer. To reduce the seeding effect and produce the 7-kDa Aβ1-40 dimers and the 13-kDa Aβ1-42 oligomers, the dissolved peptides were sonicated for three pulses of 10 seconds using a Vibra Cell VCX130 (Sonics and Material Inc.) with a 6 mm tip. The incubation times for the 7-kDa Aβ1-40, 13-kDa Aβ1-40 or 13-kDa Aβ1-42, 70-kDa Aβ1-42 and 70-kDa Aβ1-40 and >200-kDa Aβ1-42 were 15 minutes, 30 minutes, 1 hour, 18 hours and 2 hours, respectively. To fragment the >200-kDa Aβ1-42 species, each 1 ml aliquot was sonicated for three pulses of 4 seconds at 60% of maximum amplitude. The aliquots were pooled and then divided again to ensure uniformity between the aliquots. All of the size ranges of the Aβ oligomers were reproducible. However, the optimal incubation times to produce each type of oligomer had to be determined empirically each time the oligomers were prepared. Peptides were frozen in aliquots of 1 ml at −20°C.

### Effect on sAPPα and sAPPβ production

B103 rat neuroblastoma cells and 7W-CHO cells were seeded at 100,000 or 50,000 cells per well in a 96-well plate and allowed to grow for one day. The B103 rat neuroblastoma cells stably expressing human wild-type APP were a kind gift from Dr. Lennart Mucke (J. David Gladstone Institute of Neurological Disease and University of California, San Francisco). The 7W-CHO cells were a kind gift from Dr. Edward Koo (Department of Molecular Pathology, University of California, San Diego). The cell culture medium was then changed and the cells treated with 1 µM disulfiram, 1 µM sulfiram, or 0.01% DMSO (control). After 24 hours, the amount of sAPPα and sAPPβ produced in the medium was quantified using the AL231C and AL232C AlphaLISA kits from Perkin Elmer, respectively, modified using a custom biotinylated APP antibody.

### Effect on ADAM10 cleavage in vitro

MBP-eAPP_230–624_ was incubated with 1 uM disulfiram for 15 minutes. Then, ADAM10 was added for 60 minutes. Time points were taken every 20 minutes. The cleavage of MBP-eAPP_230–624_ by the enzyme was quantified by the appearance of a MBP-eAPP_230–624_ fragment detected by a sAPPα AlphaLISA assay kit (Perkin Elmer, AL254C) using the PE Enspire reader.

### Fluorescence studies

The thioredoxin fusion protein containing residues 290–624 of APP_695_ (TRX-eAPP_290–624_), the maltose binding protein fusion protein containing residues 230–624 (MBP-eAPP_230–624_) and a fragment of the APP containing residues 230–624 (eAPP_230–624_) were expressed and purified as previously described [18]. The absorption spectra of eAPP_230–624_ in the presence and absence of disulfiram, sulfiram, and 1,8-ANS were measured with a Spectramax 190 spectrophotometer (Molecular Devices) from 260 nm to 450 nm. The excitation spectra of the intrinsic tryptophan fluorescence were recorded using either a Shimadzu RF-530PC (Shimadzu Scientific Instruments) or a Spectramax XPS spectrophotometer (Molecular Devices).

Full emission spectra were measured with the Shimadzu RF-530PC using a 3 ml quartz cuvette, a 5 nm slit width and protein concentrations between 0.2–0.6 µM. To minimize dilution effects, titrations with 1,8-ANS, sulfiram, disulfiram, and the RERMS peptide, were performed with concentrated compound so that the final concentration was greater than 95% of the starting concentration. Within this dilution range, control titrations with protein and buffer indicated that the emission spectra scaled linearly with protein concentration. For the Aβ peptides, the titrations were performed so that the final concentration was greater than 75% of the starting concentration. For these larger dilutions, control titrations with the protein alone were used to generate a scaling matrix where the scale factor was both a function of wavelength and dilution. Within the peak, the scale factors for each wavelength were linear with respect to dilution. A similar scaling matrix was used to correct for the effect of dilution with DMSO. No significant changes in peak shape were observed with up to 3% DMSO, although there was a modest increase in the fluorescence intensity (<10%). All titrations were performed at 24°C. The oligomers were stored on ice during the experiment.

In addition to the titration experiments to control for possible effects that the dilution may have had on the equilibrium of the Aβ oligomers, 47 µM Aβ oligomers were incubated with 7.8 µM purified eAPP_230–624_ for 1 hour on ice before analysis. These concentrations are analogous to concentrations used to create complexes for the small angle x-ray scattering analysis. The incubated material was diluted with PBS so that the final protein concentration was 0.5 µM. The spectra were recorded with the Shimadzu RF-503PC.

For experiments with the Spectromax XPS plate reader, an excitation wavelength of 295 nm was used. This excitation wavelength was chosen to minimize both the contribution of tyrosine in the protein and the background fluorescence of the 96-well plates (Costar #3915). The quenching experiments were performed by diluting 20 µl of 0.5 mg/ml eAPP_230–624_ in 20 mM Tris pH 7.4 with varying concentrations of 1,8-ANS in DPBS plus 2% DMSO or Aβ oligomers in DPBS for a total volume of 190 µl. As with the titration experiments, the Aβ peptides did not exhibit significant fluorescence when excited at 295 nm. To correct for background, measurements were made of the various solutions in the absence of the eAPP_230–624_. Since there was no significant difference due to concentration in any of these solutions, the measurements were averaged and then subtracted from the measurements for the analogous protein-containing solutions. The concentration ranges for the peptides or compounds were chosen so that there were no apparent concentration-dependent absorption effects as shown by lack of variation in the plate blanks.

For the quenching experiments, the data were analyzed by constructing Stern-Volmer plots, F_0_/F vs. Q, where F_o_ is the intensity of the fluorescence at zero concentration, F is the intensity of the fluorescence, and Q is the concentration of the quenching agent. When quenching is through either a static (binding) or dynamic (collisional) mechanism this plot is linear and follows the equation:
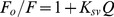
(1)where K_sv_ is the quenching constant. When both processes are present the Stern-Volmer plot is quadratic:
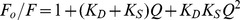
(2)where K_D_ is the equilibrium constant associated with dynamic quenching and K_s_ is the equilibrium constant associated with static quenching. All parameters were determined using PRISM (GraphPad) and the appropriate non-linear curve fitting routine.

The centroid wavelength (λ_c_) was calculated as:
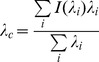
(3)where I(λ_i_) is the intensity observed for the wavelength (λ_i_). The range of wavelengths was 310–390 nm.

### Small-angle X-ray scattering (SAXS)

#### Preparation of samples

For the Aβ fragments, disulfiram, and sulfiram incubated with MBP-eAPP_230–624_, 150 µl of protein (≈0.5 mg/ml) was diluted with 400 µl of peptide or compound solution. The samples were incubated on ice for 20 minutes and then concentrated to 50 µl with 10-kDa cutoff concentrators (Millipore). All of the peptides and small molecules prepared in this manner were less than 5-kDa in molecular weight. Samples of the filtrates were used for buffer subtraction. To create a control for the disulfiram and sulfiram experiments, which contained 2% DMSO in the PBS, 400 µl of 2% DMSO in PBS was added to 150 µl of MBP-eAPP_230–624_ and then treated as the other samples.

For the other experiments, the appropriate eAPP fragment was incubated on ice with either Aβ oligomers or Aβ fragments under conditions where the peptide concentration was less than 10 µM. The samples were then concentrated and purified with a Superdex S200 size-exclusion column with 20 mM Tris pH 7.4, 100 mM sodium chloride and 2 mM EDTA. Samples of the protein buffer from the size-exclusion columns were used for buffer subtraction. Previous experiments indicated that this method of preparation resulted in Aβ oligomer-eAPP_230–624_ complexes that were stable for at least eighteen hours [18].

#### Data collection and processing

Small-angle X-ray scattering data were collected using protein concentrations in the range of 0.5–3 mg/ml and an X-ray wavelength of 1.11 Å at beam line 12.3.1 (Advanced Light Source). Data were integrated with software customized for the beam line and processed with the program PRIMUS [30]. The program GNOM [31] was used to calculate the maximum dimension and the radius of gyration and to estimate the intensity of the scattering at zero angle for higher concentration samples. The dimensional data for each sample are summarized in Table 1. Although dilutions of each sample were analyzed to concentrations of approximately 0.3 mg/ml, no significant differences were observed in the dimensional data across the concentration ranges shown in Table 1.

**Table 1:** SAXS analysis of eAPP fragments and their complexes.

Protein	Peptide	D_max_ (Å)	R_g_ (Å)	MW_calc_/MW_seq_
TRX-eAPP_290–624_	-na-	150	41	2.0
TRX-eAPP_290–624_	Aβ11-17	130	38	1.2
TRX-eAPP_290–624_	Aβ 1-28	140	42	1.5
TRX-eAPP_290–624_	APP319-335	140	41	1.3
TRX-eAPP_290–624_	7-kDa Aβ1-40	160	40	1.1
TRX-eAPP_290–624_	20-kDa Aβ1-42	160	43	2.5
eAPP_230–624_	-na-	150	44	2.0
eAPP_230–624_	RERMS	130	39	1.8
eAPP_230–624_	Aβ 1-28	170	46	0.9
eAPP_230–624_	7-kDa Aβ1-40	180	49	1.2
eAPP_230–624_	20-kDa Aβ1-42	170	46	2.6
MBP-eAPP_230–624_	-na-	190	55	2.0
MBP-eAPP_230–624_	2% DMSO	180	54	2.0
MBP-eAPP_230–624_	1,8-ANS	190	63	1.9
MBP-eAPP_230–624_	Sulfiram	170	53	1.7
MBP-eAPP_230–624_	Disulfiram	160	51	1.5
MBP-eAPP_230–624_	Aβ10-20	160	51	1.3
MBP-eAPP_230–624_	Aβ5-14	180	54	1.6
MBP-eAPP_230–624_	Aβ12-24	180	54	1.8
MBP-eAPP_230–624_	Aβ1-28	180	54	1.2
MBP-eAPP_230–624_	7-kDa Aβ1-40	210	63	1.1
MBP-eAPP_230–624_	20-kDa Aβ1-42	210	58	2.2

The maximum estimated error in the radius of gyration (R _g_) is ±2 Å. The maximum estimated error in maximum dimension (D_max_) is ±10 Å. Both values were calculated with the program GNOM [31]. The relative mass was calculated as the ratio of the apparent mass of the protein (MW_calc_) to the expected mass derived from the protein sequence (MW_seq_). The molecular weight derived from the protein sequence is 52-kDa for TRX-eAPP_290–624_, 45-kDa for eAPP_230–624_, and 90-kDa for MBP-eAPP_230–624_. For globular, non-interacting proteins, the apparent mass can be estimated by comparing the extrapolated scattering of the sample at zero scattering angle (I(0)) to that of the reference protein albumin and the eAPP fragment dimer
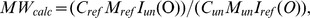
where subscripts *un* and *ref* refer to the sample and the reference protein [56]. The error is the calculated mass is primarily due to the uncertainty in the estimation of the concentrations. The maximum error was determined experimentally by replicates of within a much larger set of experiments [18] to be ±0.2, where the maximum error determines the range of values for which p<0.05. Monomeric proteins with 20–30% of their residues in random coil conformation, as expected for monomeric APP, have relative mass estimates around 1.3^51^.

## Results

To explore the utility of using the intrinsic fluorescence of the eAPP_230–624_ fragment to monitor binding of compounds, we first characterized the intrinsic fluorescence of eAPP_230–624_, probed the accessibility of the tryptophans to quenching by 1,8-ANS and then the effect of the binding of known ligands to eAPP_230–624_ on the intrinsic fluorescence. The conformational effects of the binding of these ligands, different types of Aβ oligomers and Aβ fragments, has been extensively characterized using SAXS [18]. Finally, we used a combination of intrinsic fluorescence and SAXS to determine whether molecules that alter the relative rates of α- and β- cleavage of APP were ligands of eAPP_230–624_ and whether their binding affects the dimerization of the APP ectodomain.

**Figure 1:**
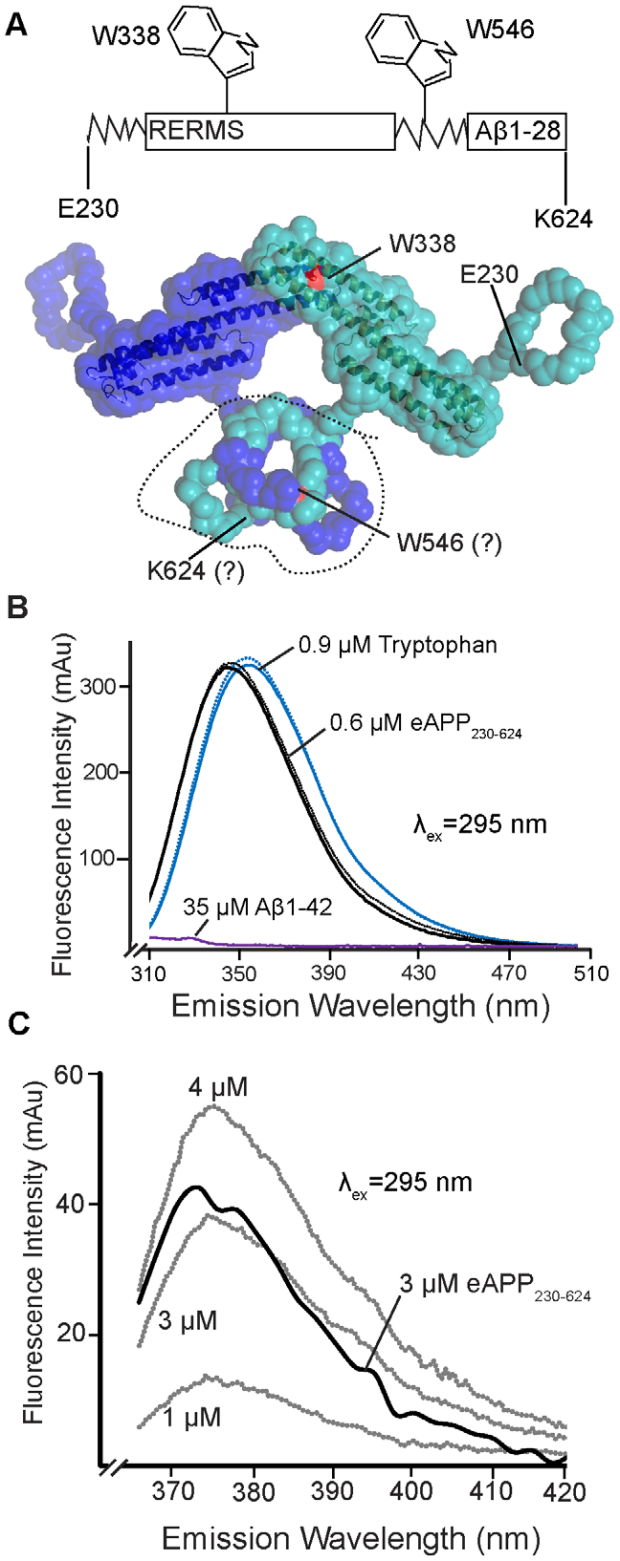
The intrinsic fluorescence of eAPP_230–624_. A) Model of the eAPP_230–624_ dimer derived from the SAXS model of eAPP_19–624_ [18] illustrating the location of the tryptophans in eAPP_230–624_ with respect to the dimerization interfaces. One monomer is shown in cyan and the second monomer is shown in blue. The surface corresponding to W338 is colored red. The corresponding residue in the blue monomer is on the back face, hidden in this view. The location of W546 and K624 cannot be precisely determined because of the low resolution of the model (20 Å). The two residues are located within the volume encircled by the dashed line. B) Emission peak of 0.64 µM eAPP_230–624_ in PBS (black solid) and PBS containing 3.3% DMSO (black dotted) obtained with an excitation wavelength (λ_ex_) of 295 nm using a Shimadzu RF-530PC spectrophotometer with a 4 ml quartz cuvette. For comparison, the spectra of 9 µM tryptophan in PBS (blue solid) and PBS-containing 3.3% DMSO (blue dotted) and 12 µM Aβ1-42 13-kDa oligomers in PBS (purple) are shown. C) Emission peak of 3 µM eAPP_230–624_ in PBS obtained with an excitation wavelength (λ_ex_) of 295 nm using a Spectramax XPS spectrophotometer (black). For reference, the emission spectra of 1–4 µM of tryptophan are also shown (grey). The peak shown is created by the design of the instrument, which displays reduced sensitivity as the wavelength approaches 360 nm.

The eAPP_230–624_ fragment was chosen for these studies because our previous study of the binding of Aβ oligomers and Aβ dimers to the ectodomain of APP established that only residues 230–624 were required for these conformational changes induced by Aβ-binding [18]. Although the primary binding site of the Aβ was determined to be within the Aβ-cognate region, significant conformational changes occur in the dimer of the ectodomain of APP (eAPP_19–624_) upon Aβ binding. Binding of both Aβ oligomers and dimers was associated with unfolding of the acidic region between the E1 (residues 19–210) and E2 (residues 290–540) domains. Binding of Aβ dimers also reduced the eAPP_19–624_ dimers to monomers, with a concomitant unfolding of residues 575–624 in addition to the acidic region. For both the Aβ oligomers and the Aβ dimers, the E1 domain was not required for either Aβ binding or dimerization. The 230–624 fragment contains only two tryptophans, W338 and W546 which simplifies interpretation of its fluorescence. The SAXS model of the homodimer of eAPP_19–624_ (Fig. 1A) predicts that the homodimer of eAPP_230–624_ will have two populations of tryptophans: W338, which should be partially solvent accessible on the surface of the E2 domain, and W546, which should be located near the C-terminal dimer interface [18]. Because of the low resolution of the SAXS model, no predictions can be made about the accessibility of W546.

### Characterization of the intrinsic fluorescence of eAPP_230–624_

eAPP_230–624_ has seven tyrosines, nine phenylalanines, and two tryptophans. Although the fluorescence of tyrosine and phenylalanine are relatively insensitive to their environment, the fluorescence of tryptophan is highly sensitive to the environment. The fluorescence peaks of tryptophans in proteins can be highly variable, between 330 nm for tryptophans in less polar environments, such as buried within a protein, and 360 nm for tryptophans in very polar environments [27]. Therefore, to obtain the intrinsic fluorescence spectrum most sensitive to conformational changes, it is useful to minimize the fluorescence from the tyrosines and phenylalanines. Using an excitation wavelength greater than 290 nm minimizes the contribution of the tyrosines and phenylalanines, since the excitation peaks of tyrosines and phenylalanines are 274 and 260 nm, respectively [32]. This principle is shown in practice for the 13-kDa Aβ1-42 oligomers, which lack tryptophan but have a modest intrinsic fluorescence due to tyrosines and phenylalanines with an excitation wavelength of 280 nm, but almost no intrinsic fluorescence at an excitation wavelength of 295 nm (Fig. 1B).

The intrinsic fluorescence spectrum of eAPP_230–624_ has a peak at 345 nm. This peak has the same peak shape as similar concentrations of tryptophan in phosphate-buffered saline (PBS), but is blue-shifted by 5 nm to 340 nm, indicating that both tryptophans are likely to be partially accessible (Fig. 1B). Because of the design of the Spectramax XPS plate reader, only wavelengths in roughly the upper third of the intrinsic fluorescence peak can be measured. The observed peak at 375 nm for tryptophan and eAPP_230–624_ is created by the design of the instrument since it is less sensitive near its emission cutoff of 360 nm (Fig. 1C).

### Accessibility of the tryptophans in eAPP_230–624_ to quenching by 1,8-ANS

**Figure 2:**
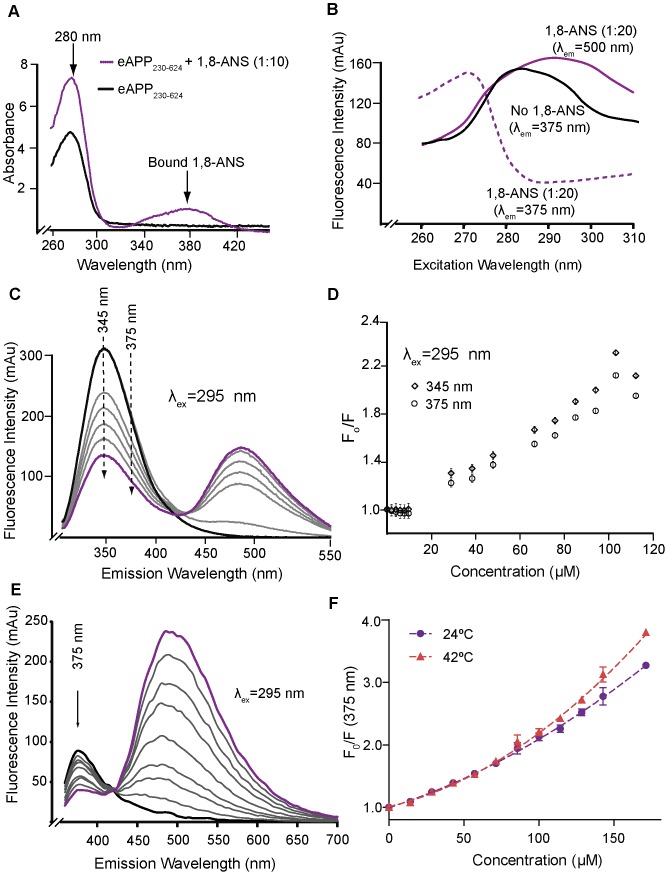
1,8-ANS binds to eAPP_230–624_. A) Difference absorption spectra of eAPP_230–624_ in the presence of 1,8-ANS (purple) at a 1∶10 molar ratio. For each difference curve, the absorption spectra of PBS, PBS plus 1,8-ANS was subtracted from the absorption spectra of 3 µM eAPP_230–624_ in the same buffer. The appearance of significant changes in the peak absorption near 280 is consistent with perturbations in the environment of some of the aromatic residues in eAPP_230–624_ caused by the binding of 1,8-ANS. The broad absorption peak centered on 380 nm is characteristic of 1,8-ANS bound to protein [55]. B) Comparison of the excitation peak for the intrinsic fluorescence at 375 nm for 3 µM eAPP_230–624_ alone (solid black) and in the presence of 1,8-ANS at molar ratio of 1∶20 protein to 1,8-ANS at 375 nm (dotted purple) and 500 nm (purple). This comparison shows that the presence of 1,8-ANS at a 1∶20 molar ratio is sufficient to redirect the absorbed photons in the region dominated by tryptophan absorption (285–300 nm) into emission peak of 1,8-ANS at 500 nm consistent with FRET between the tryptophans and bound 1,8-ANS molecules. C) Emission spectra of the titration of 0.64 µM eAPP_230–624_ with 1,8-ANS from 0–120 µM (1∶0–1∶180 molar ratio) using the Shimadzu RF-530PC. The black line indicates the starting concentration and the purple line indicates the final concentration. D) Stern-Volmer plot for intermolecular quenching of the intrinsic fluorescence of eAPP_230–624_ by 1,8-ANS at 24°C derived from titration data from the Shimadzu RF-530PC. E) Emission spectrum of 4 µM eAPP_230–624_ in PBS with varying molar ratios of 1,8-ANS from 1∶0 (black) to 1∶55 (purple) obtained using the Spectramax XPS. F) Stern-Volmer plot for intermolecular quenching of the intrinsic fluorescence of eAPP_230–624_ by 1,8-ANS at 24°C (purple) and 42°C (red) as measured on the Spectramax XPS. The protein concentration was 2 µM for E–F.

Because the accessibility of W546 is unknown, 1,8-ANS was used to probe the accessibility of the tryptophans in eAPP_230–624_. 1,8-ANS has been shown to bind to Aβ oligomers and prevent Aβ aggregation [33], [34]; therefore we reasoned that, since the Aβ-cognate region is involved in eAPP dimerization, 1,8-ANS might also bind to the dimer of eAPP_230–624_ sufficiently near W546 to quench its fluorescence. As shown in Fig. 2A, incubation of eAPP_230–624_ with 1,8-ANS had a significant effect on the absorption of the eAPP_230–624_ protein at a one-to-ten molar ratio. Binding of 1,8-ANS to the protein can be monitored by the appearance of an absorption peak near 380 nm. 1,8-ANS bound to protein absorbs in this region, while 1,8-ANS in solution does not [35]. Changes in the absorption spectra can also reveal effects on the environments of the tyrosines and tryptophans. In contrast to phenylalanine, whose absorption is relatively insensitive to environmental changes, the absorption of tyrosine and tryptophan are both highly sensitive to the polarity of the environment [36]. These properties make the difference absorption spectra a useful adjunct to the fluorescence studies for determining whether quenching of the fluorescence is due to binding rather than collisional processes. A compound that quenches the fluorescence primarily by collisional processes will not display significant changes in the difference absorption spectrum [32].

Binding of 1,8-ANS near tryptophans can quench tryptophan fluorescence through Förster energy resonance transfer (FRET). Binding of 1,8-ANS to a protein requires positive charge donors to counter the negative charge of the sulfonate group, but not necessarily a large hydrophobic pocket [37]. As shown in Fig. 2B, 1,8-ANS efficiently quenched the intrinsic fluorescence due to the tryptophans (excitation wavelengths greater than 285 nm) at a 1∶20 protein to 1,8-ANS molar ratio. Figures 2C and 2D compare the quenching observed with the Shimadzu RF-530PC (Fig. 2C) and the Spectramax XPS plate reader (Fig. 2D). The calculated quenching constants using the linear parts of the curves and equation 1 are 9.3±0.1 mM^−1^ (345 nm) and 10.9±0.2 mM^−1^ (375 nm) for the Shimadzu RF-530PC and 9.6±0.3 mM^−1^ (375 nm) for the Spectramax XPS plate reader. At higher concentrations, the Stern-Volmer plot for the quenching by 1,8-ANS is quadratic, which suggests quenching by both binding and collisional processes at high concentration (Fig. 2C). The upward shift of the curve with temperature increase is consistent with additional quenching of the intrinsic fluorescence by collisional processes at higher concentrations. However, SAXS analysis indicated a significant increase in the radius of gyration for the MBP-eAPP_230–624_ dimer incubated with a 1∶20 molar ratio of protein to 1,8-ANS similar to that seen upon binding of Aβ oligomers, indicating that binding of 1,8-ANS induces a conformational change in eAPP_230–624_ (Table 1). Therefore, conformational changes induced by increased 1,8-ANS binding may also contribute to the shape of the curve. The significant quenching below 50 µM (1∶14 protein to 1,8-ANS molar ratio) is most likely due to binding of the 1,8-ANS to the protein and the resulting FRET with nearby tryptophans. Since the fluorescence intensity drops by more than 50%, this suggests that at least two of the four tryptophans in the dimer are close to the bound 1,8-ANS. This is consistent with the hypothesis that the 1,8-ANS binds near the C-terminal dimer interface which contains two of the tryptophans and the Aβ-cognate regions known to bind 1,8-ANS. The fact that a sufficiently high concentration of 1,8-ANS (greater than 150 µM) can quench nearly all the fluorescence indicates that all four tryptophans are solvent accessible.

### Effect of Aβ binding on the intrinsic fluorescence of eAPP_230–624_

**Figure 3:**
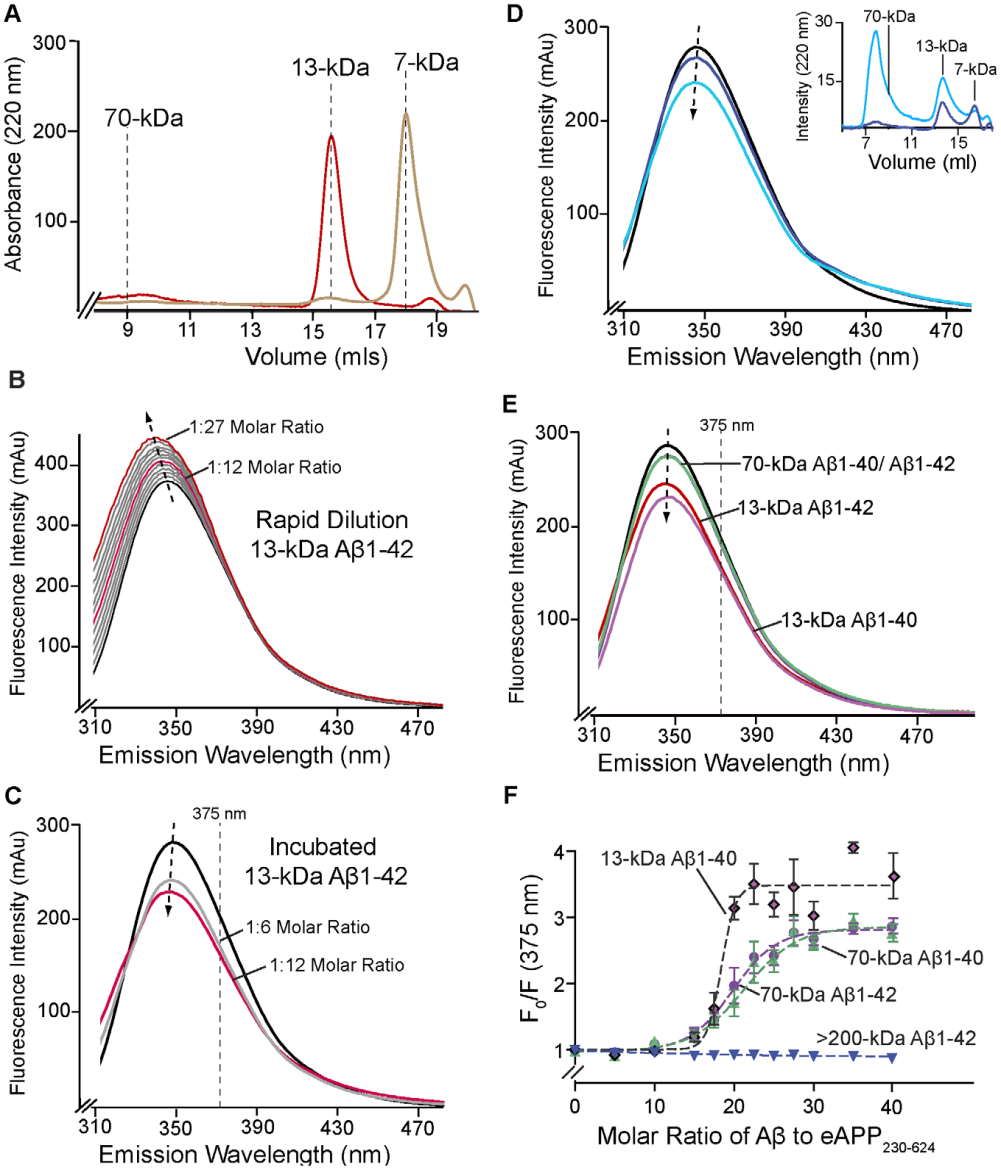
Aβ-binding to eAPP_230–624_ can be measured using intrinsic fluorescence. A) Size-exclusion chromatography analysis of Aβ oligomer preparations using a Superdex 75 column and PBS as the running buffer. As an example, 13-kDa Aβ1-42 (red) and 7-kDa Aβ1-40 (gold) are shown. B) Emission spectra of the titration of 0.64 µM eAPP_230–624_ with 13-kDa Aβ1-42 from 0–13 µM (1∶0–1∶27 molar ratio) using rapid dilution and the Shimadzu RF-530PC. The aliquots were 100 µl each diluted into a starting volume of 3 mls. The black line indicates the starting concentration and the red line indicates the final concentration. The pink line indicates a molar ratio of 1∶12 protein to Aβ oligomers. The arrow indicates the direction of shift of the fluorescence peak. C) Emission spectra of the 0.5 µM eAPP_230–624_ incubated with a 1∶6 (grey) and 1∶12 (red) molar ratio of 13-kDa Aβ1-42 oligomers. The emission spectrum of eAPP_230–624_ alone is shown in black. D) Emission spectra of 0.5 µM eAPP_230–624_ with Aβ species greater than 200-kDa before sonication (dark blue) and after sonication (light blue). The inset compares the chromatogram of 0.1 µg Aβ species before (dark blue) and after sonication (light blue) using a calibrated Superdex 75 column with PBS as the running buffer. Before sonication, the Aβ oligomers do not enter the column. E) Emission spectra of the 0.5 µM eAPP_230–624_ incubated with a 1∶6 molar ratio of 13-kDa Aβ1-40 (lavender), 13-kDa Aβ1-42 (red), 70-kDa Aβ1-40 (green) and 70-kDa Aβ1-42 (purple). The spectra of the 70-kDa Aβ1-40 and Aβ1-42 are nearly completely superimposed, so that only the 70-kDa Aβ1-40 is visible F) Changes in the intrinsic fluorescence of eAPP_230–624_ resulting from incubation of with the same Aβ oligomers from D and E:13-kDa Aβ1-40 (black with lavender center), 70-kDa Aβ1-40 (green), 70-kDa Aβ1-42 (purple), >200-kDa Aβ1-42. The dominant sizes of the Aβ oligomers were determined by size-exclusion chromatography. None of the oligomers were purified, so the size represents only the major species in the mixture. The excitation wavelength was 295 nm for all spectra and the data were measured using the Spectramax XPS.

**Figure 4:**
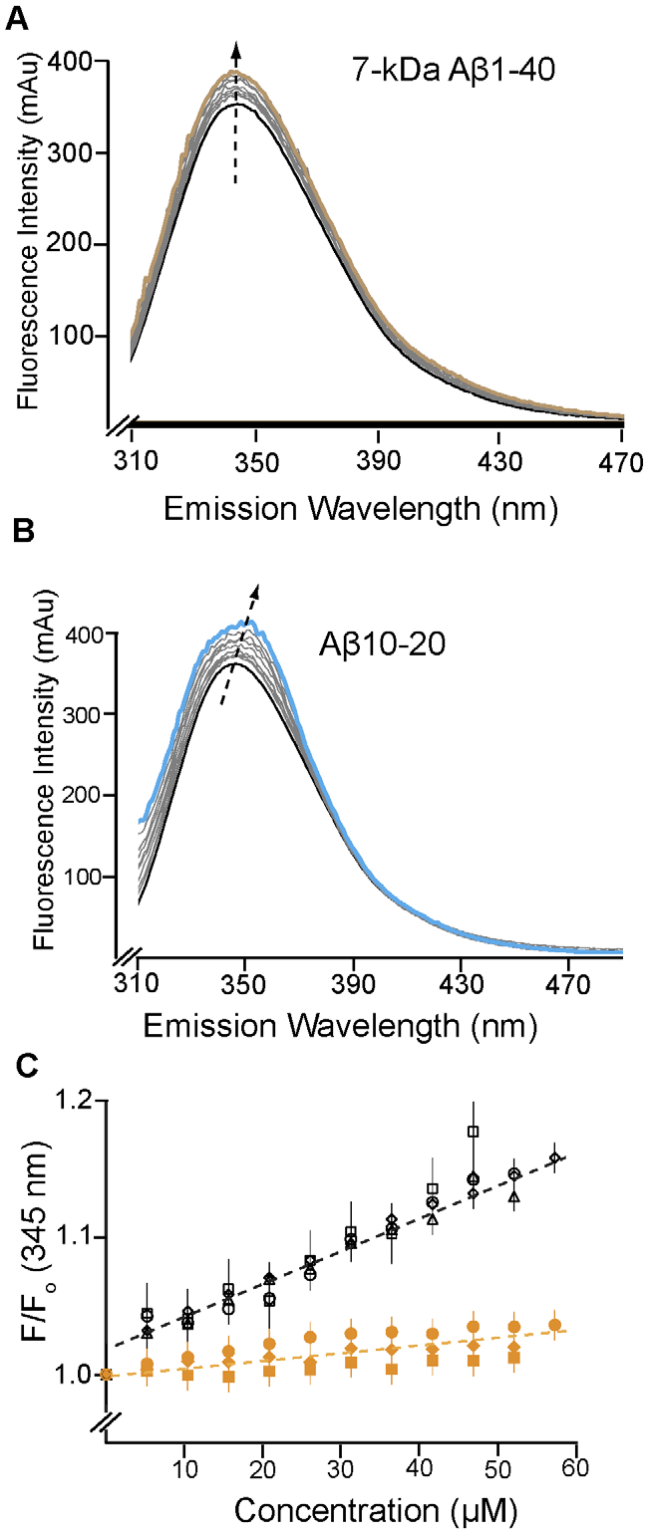
Binding of Aβ fragments to eAPP_230–624_ can be measured using intrinsic fluorescence. A) Emission spectra from the titration of 0.64 µM eAPP_230–624_ with 7-kDa Aβ 1-40 from 0–13 µM (1∶0–1∶20 molar ratio) using the Shimadzu RF-530PC. The black line indicates the starting concentration and the gold line indicates the final concentration. The arrow indicates the direction of shift of the fluorescence peak. B) Emission spectra from the titration of 0.64 µM eAPP_230–624_ with Aβ 10–20 from 0–46 µM (1∶0–1∶70 molar ratio) using the Shimadzu RF-530PC. The black line indicates the starting concentration and the blue line indicates the final concentration. The arrow indicates the direction of shift of the fluorescence peak. C) Summary of the results of titrating 0.64 µM eAPP_230–624_ with various fragments of Aβ that inhibit eAPP dimerization: Aβ1-40 (black circles), Aβ1-28 (black diamonds), Aβ12-28 (black triangles), Aβ 10–20 (black squares), Aβ12-24 (yellow squares), Aβ5-14 (yellow diamonds) and Aβ16-20 (yellow circles) from 0–60 µM (1∶0–1∶85 molar ratio depending on the fragment). The excitation wavelength is 295 nm and the emission wavelength is 345 nm. To exclude the possibility that Aβ1-28 or Aβ10-20 formed oligomers, samples of these peptides were analyzed using size-exclusion chromatography as shown in Fig. 3A. Both of these peptides eluted 0.5 mls later than the 7-kDa peak, indicating that they are smaller dimers or monomers.

To characterize the effect of the binding of Aβ oligomers on the intrinsic fluorescence of eAPP_230–624_, pools of Aβ oligomers of varying sizes were generated and classified by the size of the dominant oligomer using a calibrated Superdex-75 or Superdex-200 size-exclusion column, as described in Libeu et al (2011) [18]. Fig. 3A shows the results of the preparation of 13-kDa Aβ1-42 and 7-kDa Aβ1-40. Previous cross-linking analysis demonstrated that these peaks contain predominately trimers and dimers, respectively [18], [38]. Titration using rapid dilution with 13-kDa Aβ-42 oligomers to a final 1∶27 molar ratio dramatically shifted the emission peak from 345 to 338 nm, the centroid wavelength from 350 to 346 nm, and increased the intensity of the fluorescence by 114%. This pattern in the change of fluorescence is consistent with a decrease in the accessibility of one or more tryptophans (Fig. 3B). However, the pattern of change in the intrinsic fluorescence is highly similar to the change observed for titration with Aβ1-40 dimers (Fig. 4A). The similarity suggests that the changes in the intrinsic fluorescence might be induced by binding of Aβ1-42 dimers or monomers rather than oligomers. Several effects may contribute to this observation. Oligomerization of larger Aβ oligomers has been reported to be reversible by dilution at similar concentrations [39]. Aβ1-42 dimers are known to be in equilibrium with Aβ1-42 trimers [38]. Finally, the sheering forces induced by the constant stirring may retard the formation of larger oligomers or cause larger oligomers to break-up.

To determine whether the order of dilution was important, the same 13-kDa Aβ1-42 oligomers were incubated with eAPP_230–624_ on ice prior to dilution. As shown in Fig. 3C, the pattern of the change in the fluorescence with increasing oligomer concentration was strikingly different. At the 1∶12 molar ratio, the fluorescence intensity was 82% of eAPP_230–624_ alone; while for the rapid dilution, the fluorescence intensity was 108% of eAPP_230–624_ alone. The shift in the peak wavelengths and the centroid wavelengths is similar between the two methods, from 345 nm to 343 nm for the peak wavelength and from 350 nm to 348 nm for the centroid wavelength. Since the total protein and Aβ concentrations are similar between the two experiments, the difference is not driven by final concentrations or non-specific solvating effects due to the presence of the Aβ. Analysis with small angle x-ray scattering (SAXS) of the complexes formed during incubation of eAPP_230–624_ or MBP-eAPP_230–624_ with similar-sized Aβ1-42 oligomers disclosed a molecular mass consistent with the binding of an approximately 25–30-kDa Aβ1-42 oligomer to a dimer of the protein. This observation suggests that the fluorescence spectra obtained from incubating the protein with the oligomers prior to dilution corresponds to the binding of the 25–30-kDa Aβ1-42 oligomer to eAPP_230–624_ and that the large decrease in fluorescence intensity after incubation is due to quenching of one of the tryptophans as a consequence of this binding.

As we have reported previously [18], the apparent size of the oligomer that binds to the eAPP fragments is independent of the size of the oligomers incubated with the fragment as long as the oligomer preparations contain oligomers between 13-kDa and 70-kDa and A-11 positive oligomers are present. This observation suggests that during the incubation a remodeling process occurs that produces oligomers of the particular size that preferentially bind to eAPP. To determine whether the remodeling process could be detected by intrinsic fluorescence, we incubated the eAPP_230–624_ with Aβ oligomers with different characteristic sizes. As shown in Fig. 3D, incubation of eAPP_230–624_ with Aβ1-42 species greater than 200-kDa in size at a 1∶6 molar ratio gave a relatively minor change in the fluorescence spectra consistent with the poor ability of the larger species to make complexes with the eAPP_230–624_ observed previously. Sonication of these large Aβ species broke them into smaller, more soluble oligomers (Fig. 3D inset). Incubation of eAPP_230–624_ with the sonicated Aβ1-42 material produced a decrease in the fluorescence intensity to 87% that of eAPP_230–624_ and a 1 nm blue-shift in the centroid wavelength, from 250 nm to 249 nm. Similar modest shifts were observed with the incubations with 70-kDa Aβ1-40 and 70-kDa Aβ1-42 at the 1∶6 molar ratio (Fig. 3E). Like the 13-kDa Aβ1-42 (Fig. 3C), 13-kDa Aβ1-40 decreased the intensity to 80%, however it did not produce a significant blue shift.

Although the Shimadzu RF-503PC gives the most information about the tryptophan environments, monitoring the binding at higher molar ratios is limited by the large amount of oligomeric Aβ required to record high-quality spectra. As shown with the comparison between the two instruments for quenching with 1,8-ANS, the Spectramax XPS gives relatively little information on the peak shape, but it can be used to monitor binding with a 30-fold lower investment of Aβ oligomers because of the symmetrical nature of the peaks. We found that studies could be done using concentrations of Aβ up to 120 µM in which all the replicates can be measured at the same time within 15 minutes of setup. These characteristics are highly advantageous for the higher concentrations of Aβ, which continue to aggregate at 24°C, enhancing reproducibility of the measurements. As shown in Fig. 3F, increasing the molar ratio with either the Aβ1-42 or Aβ1-40 oligomers from Fig. 3E resulted in further quenching of the fluorescence at 375 nm, presumably due to similar shifts in the fluorescence peak shape to those shown in Fig. 3C. The efficiency of the quenching by the Aβ oligomers depended on the size of the oligomers. The largest Aβ-species (greater than 200-kDa) gave a slight enhancement of the intrinsic fluorescence similar to the curves shown for the Aβ fragments in Fig. 4D. The half-maximum point of each curve is close to the 1∶20 molar ratio which was shown to be effective at inducing conformational changes in the ectodomain of APP [18]. The similarity supports the conclusion that co-incubation of Aβ oligomers with eAPP_230–624_ promotes a remodeling process that results in the preferred approximately 30-kDa size for binding to the ectodomain of APP. The sigmoidal shape of the titration curves for the oligomers suggested a cooperative binding process. Fitting of the curves using the program PRISM indicated that the curves could be fit by the equation
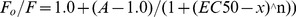
(4)where F_0_ is the fluorescence at zero concentration, x is the molar ratio of Aβ oligomers to eAPP_230–624_, n is the number of Aβ molecules bound, A is a parameter related to the maximum response of the intrinsic fluorescence, and EC50 is the molar ratio that invokes half the response. For the Aβ oligomers less than 70-kDa in size, constraining n to 7 maximized the fit for all three curves in Fig. 1C (R^2^ = 0.88 for all three curves). The log(EC50)s were 1.27±0.02, 1.32±0.01, and 1.29±0.01 for 13-kDa Aβ1-40, 70-kDa Aβ1-40, and 70-kDa Aβ1-42, respectively. The average of these EC50 values corresponds to a molar ratio of approximately 1∶20 eAPP_230–624_ to Aβ. None of the log(EC50)s were significantly different at the p<0.05 confidence level, indicating that these different Aβ oligomer preparations varied only in the amplitude of the maximal response (A). This amplitude most likely varies because each pool of oligomers varies in its ability to remodel and produce eAPP-binding oligomers. Although we do not understand the remodeling process, the ratio of the amplitudes suggests that the 13-kDa Aβ1-40 can produce about 150% more oligomers that can bind eAPP, which is consistent with the conclusion of the earlier SAXS studies that demonstrated that the pools with smaller oligomers (≈20-kDa) were more efficient at binding the ectodomain than pools with larger oligomers (≈70-kDa) [18]. A value of seven for n is in surprisingly good agreement with the number of molecules bound to the eAPP fragments as estimated by SAXS. The molecular weight, as estimated by SAXS, clusters between 25–30-kDa for the different experiments (Table 1), while the mass of an Aβ1-42 heptamer is 30-kDa.

In contrast to the Aβ oligomer pools, titration of eAPP_230–624_ with Aβ1-40 dimers (Fig. 4A) or fragments of Aβ resulted in a small but significant enhancement of the intrinsic fluorescence to 110–115% and blue shifts in the centroid wavelength between 0.5–1.5 nm. For comparison, calculation of the expected error in the centroid wavelength from the average spectra and repeated independent measurements of the eAPP_230–624_ spectra both gave a variation in the centroid wavelength of ±0.2 nm. No significant differences were observed between the results for the titration protocol and the incubation protocol for the Aβ1-40 dimers (not shown). The fragments shown in Fig. 4C, Aβ1-28, Aβ12-28, and Aβ10-20 acted like Aβ1-40 dimers in terms of the changes in the intrinsic fluorescence, except for the shortest peptide (Aβ10-20), which caused a 5 nm red-shift at the peak and a 2 nm blue shift in the centroid wavelength, indicating a significant distortion of the shape of the intrinsic fluorescence peak (Fig. 4B). The change in the normalized fluorescence for each peptide was highly similar, clustering about the same line. This uniformity is consistent with the observation that all of these peptides induce monomerization of the eAPP ectodomain and result in a similar highly flexible eAPP monomer [18]. The changes in the fluorescence spectra are quite modest as compared to the Aβ oligomers consistent with Aβ dimers and fragments being associated with monomerization of the protein (Table 1). All of the fragments that monomerized eAPP_230–624_ also have a strong binding affinity for Aβ oligomers and some activity in reducing the rate of fibrillization [40]. This correlation suggests that these peptides bind directly to the Aβ-cognate region, remote from W338. Therefore, the expected shift for the tryptophans would be an increase in the solvation of W546 due to disruption of the dimerization interface near the Aβ-cognate region, which is consistent with the modest changes observed in the fluorescence spectra. The larger changes in the fluorescence spectra observed for Aβ10-20 suggest the complex has a different conformation than the complex with the other peptides, or, alternatively, the Aβ10-20 may interact directly with one of the tryptophans. SAXS analysis of this complex indicated that the monomer produced was significantly smaller than that produced with the larger peptides, with a maximum dimension of 160 Å and a radius of gyration of 51 Å, in contrast to the 7-kDa Aβ-40 complex, which had a maximum dimension of 210 Å and a radius of gyration of 63 Å.

Our data indicate that there is a distinct pattern of change in the intrinsic fluorescence induced by Aβ oligomers (which stabilize the eAPP homodimer) vs. Aβ fragments and dimers (which monomerize the eAPP homodimer). This difference is consistent with the conclusions from the previous SAXS analysis that the final conformation of the ectodomain in each complex is very different from the native conformation of the dimer, as is the conformation of the heteromultimeric ectodomain-Aβ oligomer complex from the ectodomain-Aβ dimer complex.

Both the Aβ oligomers and the 1,8-ANS demonstrate that compounds that bind near the C-terminal dimer interface may generate a large signal in the intrinsic fluorescence. These results indicate that the intrinsic fluorescence of the eAPP_230–624_ dimer may be used to monitor binding of small molecules to eAPP_230–624_ near the C-terminal dimer interface of the ectodomain of APP.

### Compounds that bind to eAPP_230–624_ may alter the balance of α- and β- cleavage

Of particular interest is the identification of compounds that can alter the balance between α- and β- cleavage of APP, but do not affect the processing enzymes directly. A 17-amino acid peptide (APP319–335) that contains the RERMS sequence is an example of such a compound. This peptide is sufficient to stimulate growth of fibroblasts in an APP-independent manner [41] and enhances the production of sAPPα in neuronal cells [42]. The RERMS peptide itself (APP 328–332) has a similar activity, albeit with a 10-fold reduced potency. The mechanism by which the RERMS peptide enhances the production of sAPPα is unknown, although evidence suggests that the RERMS peptide binds to cell surface receptors.

**Figure 5:**
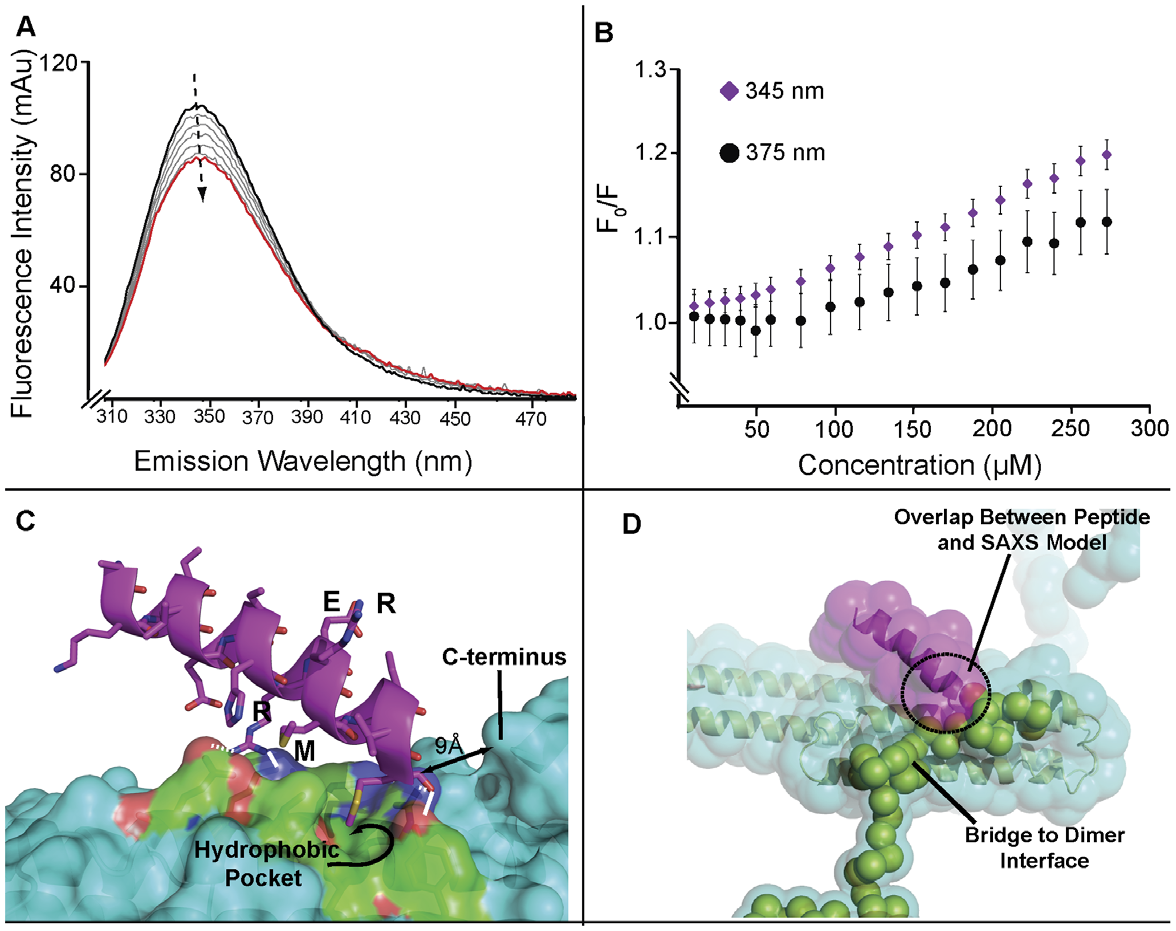
Interaction of RERMS peptides with eAPP. A) Emission spectra from the titration of 0.64 µM eAPP_230–624_ with RERMS peptides from 0–270 µM (1∶0–1∶420 molar ratio) using the Shimadzu RF-530PC. The black line indicates the starting concentration and the red line indicates the final concentration. The arrow indicates the direction of shift of the fluorescence peak. B) Stern-Volmer plot for the intermolecular quenching of 0.64 µM eAPP_230–624_ by the RERMS peptide for the emission wavelengths: 345 nm (purple diamonds) and 375 nm (black circles) C) Model of APP319-335 peptide bound to the E2 domain of APP derived from a dimer of an E2 domain fragment (residues 299 to 490) observed in its crystal structure protein data base entry 1RW6 [25]. The major interactions between the peptide and the protein are two hydrogen bonds involving Arg 328 and both polar and hydrophobic interactions between Met 335 and the hydrophobic pocket. The rim of the hydrophobic pocket into which Met 335 binds is 9 Å from the C-terminus of the E2 domain fragment. D) Superposition of the peptide model (pink) with the SAXS model. The green balls show the predicted path in the SAXS model of the chain that forms a bridge from the terminus of the E2 domain to the dimer interface containing the C-terminus of eAPP_230–624_. The overlap of the surfaces shows the overlap between the peptide docked into its binding site and the predicted path of the eAPP_230–624_ residues indicating that the two models are incompatible. This overlap suggests a mechanism by which binding of an RERMS peptide in the binding site predicted by the crystal structure could influence the stability of the dimer of eAPP_230–624_.

One of these receptors may be APP, since SAXS analysis of the complex of the peptide APP319–335 with the TRX-eAPP_290–624_ construct confirmed that the longer peptide competes with dimerization of the TRX-eAPP_290–624_ fragment (Table 1), just as Aβ1-40 dimers and Aβ fragments do. As shown in Fig. 5A, the RERMS sequence quenches the intensity of the intrinsic fluorescence with an approximately 2 nm red-shift in the peak position and a 1 nm red-shift in the centroid wavelength. The change in shape of the peak with increasing concentrations of peptide can be seen in the smaller rate of change in the fluorescence at 375 nm than near the peak (345 nm) (Fig. 5B). Analysis of the complex of eAPP_230–624_ with RERMS by SAXS indicates that the complex is a dimer with a significantly smaller maximum dimension and radius of gyration than the native dimer. The likely binding site of the RERMS peptide is not the Aβ-cognate region. In the crystal structure of the E2 domain, the RERMS sequence contributes to the formation of an E2-dimer, indicating a binding site in the E2 domain for this sequence (Fig. 5C). Superposition of this particular dimer found in the crystal structure with the dimer model derived from SAXS [18] suggests residues 319–335 from one of the subunits of the dimer from the crystal structure occupy the same space as the residues that lead to the C-terminal dimerization interface in the SAXS model (Fig. 5D). The conflict between these two models suggests that binding of RERMS-containing peptides such as APP319–335 in this location could modulate the dimerization of the APP ectodomain by altering the stability of the C-terminal dimerization interface. The shorter peptide (RERMS) is less likely to interfere with the dimerization because of its greater flexibility. The positioning of these peptides is consistent with the observation that the longer peptide forms a monomer complex, while the shorter peptide forms a more compact dimer.

**Figure 6:**
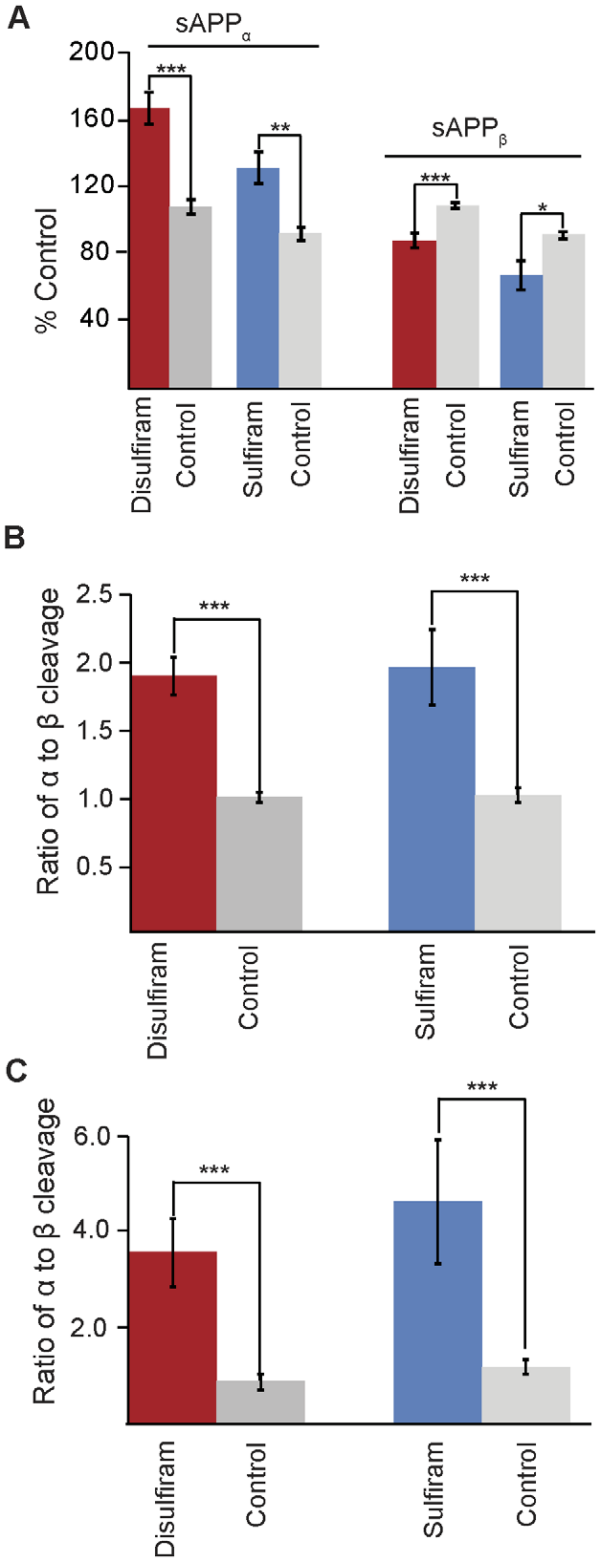
Disulfiram and Sulfiram increase sAPPα production. A) Effect of treatment with disulfiram and sulfiram on sAPPα and Aβ production in 7W-CHO cells. 7W-CHO cells were seeded at 50,000 cells per well in a 96-well plate and allowed to grow for one day. The cell culture medium was then changed and the cells treated with 1 µM disulfiram, 1 µM sulfiram, or 0.01% DMSO (control). After 24 hours, the amount of sAPPα and sAPPβ produced in the medium was quantified using the AL231C and AL232C AlphaLISA kit from Perkin Elmer, modified using a custom biotinylated human APP antibody. B) Effect on the ratio of α to β cleavage in 7W-CHO cells, as calculated by dividing the amount of sAPPα to sAPPβ produced by the 50,000 7W-CHO cells during one day under the effect of either disulfiram, sulfiram or a DMSO control. C) Effect on the ratio of α to β cleavage in B103 cells. B103 neuroblastoma cells were seeded at 100,000 cells per well in a 96-well plate and allowed to grow for one day. The cell culture medium was then changed and the cells treated with 1 µM disulfiram, 1 µM sulfiram, or 0.01% DMSO (Control). After 24 hours, the amount of sAPPα and sAPPβ produced in the medium was quantified using the same modified AL231C and AL232C AlphaLISA kit from Perkin Elmer. The ratio of α to β cleavage was calculated by dividing the amount of sAPPα to sAPPβ produced by the 100,000 B103 cells during one day under the effect of the disulfiram, sulfiram or DMSO control.

**Figure 7:**
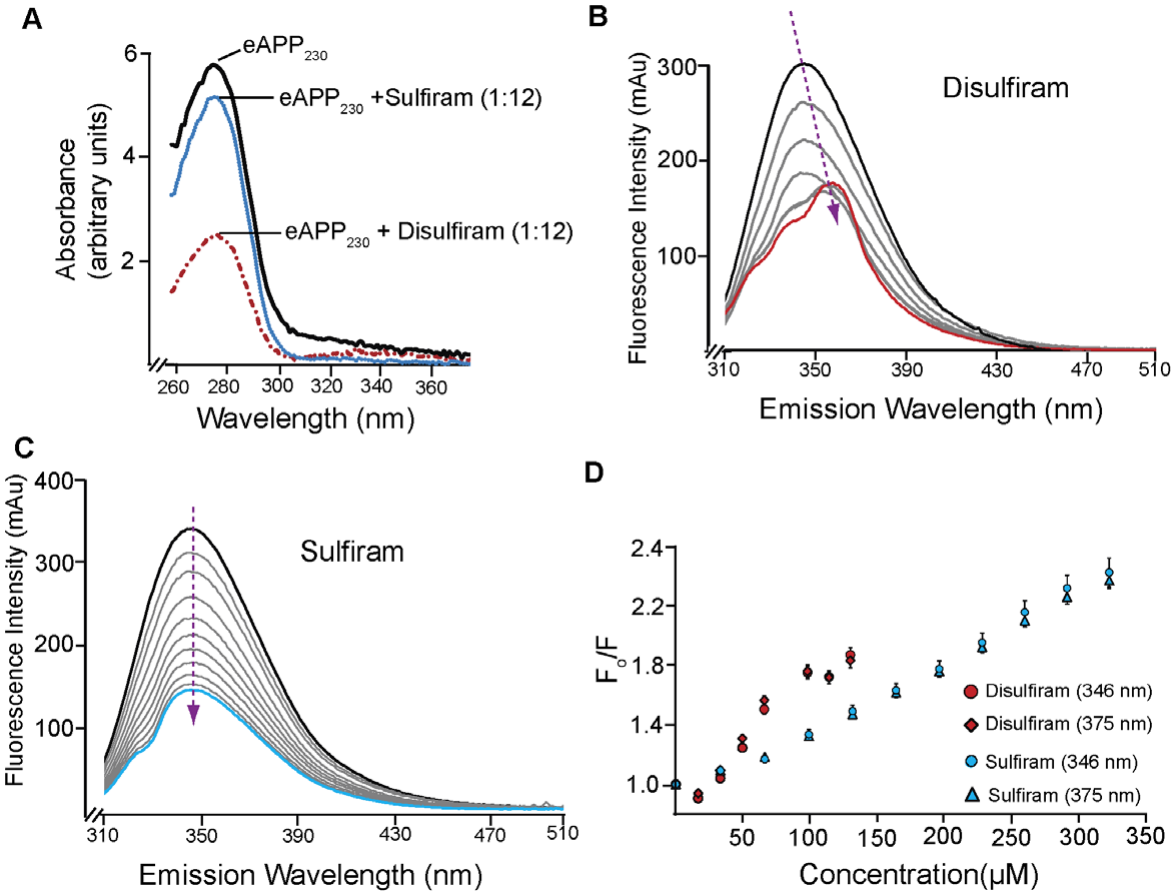
Disulfiram and Sulfiram bind to eAPP_230–624_. A) Difference absorption spectra of eAPP_230–624_ in the presence of sulfiram (blue) and disulfiram (dotted red) at a 1∶6 molar ratio. For each difference curve, the absorption spectra of PBS +2% DMSO, PBS plus 2% DMSO and 50 µM sulfiram or PBS plus 2% DMSO and 50 µM disulfiram were subtracted from the absorption spectra of 8.3 µM eAPP_230–624_ in the same buffer. B) Emission spectra from the titration of 0.64 µM eAPP_230–624_ with disulfiram from 0–130 µM (1∶0–1∶202 molar ratio) using the Shimadzu RF-530PC. The black line indicates the starting concentration and the red line indicates the final concentration. The arrow indicates the direction of shift of the fluorescence peak. C) Emission spectra from the titration of 0.64 µM eAPP_230–624_ with sulfiram from 0–320 µM (1∶0–1∶500 molar ratio) using the Shimadzu RF-530PC. The black line indicates the starting concentration and the blue line indicates the final concentration. The arrow indicates the direction of shift of the fluorescence peak. E) Stern-Volmer plot for the intermolecular quenching of 0.64 µM eAPP_230–624_ with either disulfiram (346 nm –red circles and 375 nm red diamonds) or sulfiram (346 nm–blue circles and 375 nm-blue diamonds).

Our second example emerged from a previous screen of a clinical library for compounds that alter the balance of α- and β-cleavage of APP: the compounds disulfiram (Antabuse, used to treat chronic alchoholism) and sulfiram (Temosol, an ectoparasiticide) were identified as having the ability to enhance α-cleavage significantly in 7W CHO (Fig. 6A and 6B) and B103 (Fig. 6C) cells transfected with APP. Addition of disulfiram or sulfiram to eAPP_230–624_ resulted in a significant drop in the extinction coefficient (Fig. 7A). This change was not due to aggregation of the protein caused by the addition of the DMSO. Analysis by size-exclusion chromatography indicated no significant changes in the elution time of the native dimer of eAPP_230–624_ in the presence of 2% DMSO at the concentrations used for the difference absorption and fluorescence studies (≤0.5 mg/ml) (not shown), nor was there a significant change in the size of the protein in the SAXS analysis (Table 1). Both disulfiram and sulfiram significantly quenched the intrinsic fluorescence (Fig. 7B and 7C). In addition, disulfiram significantly distorted the peak shape, shifting the fluorescence peak from 344 nm to 357 nm, indicating that at least one of the tryptophans is fully hydrated. Both the difference absorption spectrum and the fluorescence are consistent with a significant conformation change on binding of the disulfiram or the sulfiram to eAPP_230–624_. The Stern-Volmer plot for disulfiram has a slope 1.6 times greater than the slope for sulfiram (Fig. 7D) indicating that disulfiram has a higher binding affinity for eAPP_230–624_ than sulfiram.

**Figure 8:**
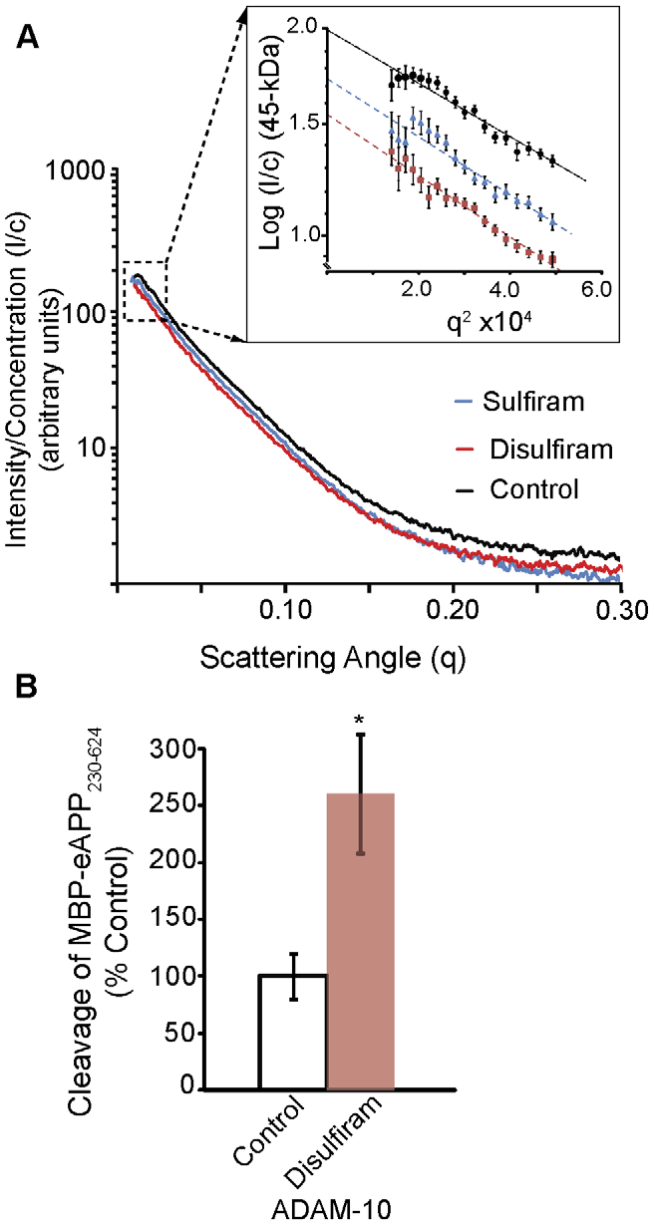
Disulfiram inhibits dimerization of MBP-eAPP_230–624_. A) Small angle x-ray scattering analysis of MBP-eAPP_230–624_ incubated with 50 µM disulfiram or 50 µM sulfiram at a 1∶12 molar ratio in PBS plus 2% DMSO. Incubation with either disulfiram or sulfiram produces significant deviations in the scattering curves indicating a significant shift in size and shape. The Guinier plot of MBP-eAPP_230–624_ (black), sulfiram-treated (blue), and disulfiram-treated (red) is shown in the inset. The y-axis of the Guinier plot is normalized so that one unit is proportional to 90-kDa (the molecular mass of MBP-eAPP_230–624_). The molecular mass of the complex can be obtained from the extrapolated y-intercept of the best-fit line. The downward shift of the line indicates that both sulfiram and disulfiram shift the monomer-dimer equilibrium in favor of the monomer. The curvature of the Guinier plot for disulfiram is evidence that the sample is a mixture of monomer and dimer. B) Cleavage of MBP-eAPP_230–624_ with ADAM10 as measured by AlphaLisa.

To determine whether binding of sulfiram or disulfiram caused significant conformational changes in the MBP-eAPP_230–624_, we examined the conformation of eAPP_230–624_ in the presence of a 12-fold molar excess of sulfiram or disulfiram by small angle x-ray scattering. The MBP-eAPP_230–624_ fusion protein was used for the SAXS analysis because both TRX-eAPP_290–624_ and eAPP_230–624_ tended to precipitate upon addition of 1–2% DMSO at the concentrations required for the analysis (>1.5 mg/ml). The presence of both sulfiram and disulfiram significantly affected the monomer-dimer equilibrium of MBP-eAPP_230–624_ in favor of the monomer, as evidenced by the decrease in apparent molecular weight (Fig. 8A). Consistent with the interpretation that disulfiram binding favors the MBP-eAPP_230–624_ monomer; the maximum dimension and radius of the disulfiram-treated MBP-eAPP_230–624_ were significantly smaller than the dimer of MBP-eAPP_230–624_ (Table 1). This monomer has a different conformation than the monomer induced by binding of the Aβ1-40 dimers, since the latter have a larger radius of gyration and maximum dimension than the MBP-eAPP_230–624_ dimers due to uncoiling of the acidic region between the two domains. The weaker effect of sulfiram in the SAXS analysis, intrinsic fluorescence, and difference absorption spectra, is consistent with its reduced ability to enhance sAPPα production in cells.

To confirm that disulfiram binding disrupts the dimer interface of the fusion protein and changes the accessibility of the α-cleavage site to proteolysis, MBP-eAPP_230–624_ was probed using ADAM10, a physiologically relevant alpha-secretase candidate protease involved in the production of sAPPα [43]. As shown in Fig. 8B, treatment with disulfiram did in fact increase the accessibility of the α-cleavage site near lysine 612 to ADAM10 cleavage.

## Discussion

Our data confirm that intrinsic fluorescence of the eAPP_230–624_ fragment is useful as a tool in the discovery of compounds or peptides that inhibit the dimerization of eAPP_230–624_ or alter the conformation of the dimer. Although it is difficult to interpret the spectra in terms of specific conformational changes because the dimer of eAPP_230–624_ contains four tryptophans, the fluorescence of those tryptophans is highly responsive to binding of Aβ oligomers or fragments to eAPP_230–624_, which induce large enough conformational changes to be detected by small angle x-ray scattering. In addition, the examples of the RERMS-containing peptides, disulfiram, and sulfiram, suggest that intrinsic fluorescence can be used to identify non-Aβ-based compounds that inhibit the dimerization of eAPP_230–624_ or alter the conformation of the dimer. These limited examples suggest that this APP conformation modulation property correlates with the ability of such compounds to influence the ratio of α- vs. β-cleavage of APP in cells. Our data suggest that these compounds may be analogous to a series of compounds that bind the APP dimerization interface within the transmembrane domain, the GXXXG motif, and have been shown to be γ-site modulators [44]. The mechanism by which sulfiram, disulfiram, and the RERMS-containing peptides are effective in increasing the α-cleavage of APP remains to be determined. We hypothesize that the activity of sulfiram and disulfiram is due to their ability to destabilize intermolecular interactions in the full-length APP dimer near the Aβ-cognate region and to alter the accessibility of the α-, β-cleavage sites to proteases. Because α-cleavage occurs primarily on the cell surface [45], compounds with this type of APP conformation modulation activity as measured by changes in the tryptophan fluorescence should favor α-cleavage over β-cleavage. Increasing the accessibility of the α-cleavage site on the cell surface is only one possible mechanism. β-cleavage is also thought to be more efficient for dimeric than monomeric APP [46], so that compounds interfering with dimerization of eAPP_230–624_ by altering the conformation near the BACE site could also alter the ratio of α-, β-cleavage. Finally, the compounds could induce conformational changes that alter APP's ability to form heterodimers or heparin-mediated homodimers. APP has been shown to interact with a number of different cell surface proteins, and some of these compete with Aβ for binding to APP. For example, competition between exogenously added Aβ1-42, which rapidly aggregates into larger oligomers, and the common neurotrophin receptor p75^NTR^, for APP binding, has been demonstrated in cell culture [47]. The large conformational change associated with oligomeric Aβ binding suggests a mechanism by which oligomeric Aβ binding could compete with other ligands, by precluding conformations of the ectodomain favorable to their binding. This type of competition may have a significant impact on both the signaling and processing of APP, since the ectodomain of APP has been implicated in forming heterodimers and higher order complexes with a number of other ligands and receptors such as Netrin-1 [13], ApoE [48], [49], Notch [50], [51], p75^NTR^ [47], APLP1, APLP2 [52], BRI2 [53], and BRI3 [54]. For all of these proteins, interaction with APP occurs through a region containing the Aβ-cognate sequence, which forms part of one of the dimerization interfaces of eAPP_230–624_. Although the functions of the British dementia proteins, BRI2 and BRI3, are unknown, the other proteins have been implicated in signal transduction complexes that regulate the balance of neurite outgrowth vs. retraction. Compounds that bind to the ectodomain of APP near this region and influence the conformation of the residues surrounding the Aβ-cognate region could influence both homo- and heterodimerization, and thereby change the rate of APP turnover or proteolysis.
